# Self-Management Support Interventions for Stroke Survivors: A Systematic Meta-Review

**DOI:** 10.1371/journal.pone.0131448

**Published:** 2015-07-23

**Authors:** Hannah L. Parke, Eleni Epiphaniou, Gemma Pearce, Stephanie J. C. Taylor, Aziz Sheikh, Chris J. Griffiths, Trish Greenhalgh, Hilary Pinnock

**Affiliations:** 1 Multidisciplinary Evidence Synthesis Hub (mEsh), Centre for Primary Care and Public Health, Blizard Institute, Barts and The London School of Medicine and Dentistry, London, United Kingdom; 2 Centre for Technology Enabled Health Research (CTEHR), Coventry University, Coventry, United Kingdom; 3 Usher Institute of Medical Informatics and Population Health Sciences, The University of Edinburgh, Edinburgh, United Kingdom; 4 Nuffield Department of Primary Care Health Sciences, Medical Sciences division, University of Oxford, Oxford, United Kingdom; Carl von Ossietzky University of Oldenburg, GERMANY

## Abstract

**Background:**

There is considerable policy interest in promoting self-management in patients with long-term conditions, but it remains uncertain whether these interventions are effective in stroke patients.

**Design:**

Systematic meta-review of the evidence for self-management support interventions with stroke survivors to inform provision of healthcare services.

**Methods:**

We searched MEDLINE, EMBASE, CINAHL, PsychINFO, AMED, BNI, Database of Abstracts of Reviews for Effectiveness, and Cochrane Database of Systematic Reviews for systematic reviews of self-management support interventions for stroke survivors. Quality was assessed using the R-AMSTAR tool, and data extracted using a customised data extraction form. We undertook a narrative synthesis of the reviews' findings.

**Results:**

From 12,400 titles we selected 13 systematic reviews (published 2003-2012) representing 101 individual trials. Although the term ‘self-management’ was rarely used, key elements of self-management support such as goal setting, action planning, and problem solving were core components of therapy rehabilitation interventions. We found high quality evidence that supported self-management in the context of therapy rehabilitation delivered soon after the stroke event resulted in short-term (< 1 year) improvements in basic and extended activities of daily living, and a reduction in poor outcomes (dependence/death). There is some evidence that rehabilitation and problem solving interventions facilitated reintegration into the community.

**Conclusions:**

Self-management terminology is rarely used in the context of stroke. However, therapy rehabilitation currently successfully delivers elements of self-management support to stroke survivors and their caregivers with improved outcomes. Future research should focus on managing the emotional, medical and social tasks of long-term survivorship.

## Introduction

The incidence of stroke continues to rise in low- and middle-income countries,[1] and although it is now declining in high-income countries, demographic changes and improved survival means the overall numbers of people living with stroke is high and likely to increase.[2] One in 20 adults in high income countries are now affected by stroke,[1] and one in three stroke survivors are left permanently disabled, placing a large burden on health and social care.[3–5]

Promotion of self-management is a core response of healthcare systems globally to the challenge of long-term condition (LTC) survivorship.[5–7] Currently, available support for self-management ranges from the provision of disease-specific information via a website or leaflet,[8] to extensive generic programmes such as the UK Expert Patient Programme, which aims to promote behavioural change by building the confidence of individuals to manage their condition and the biopsychosocial impact of living with a LTC.[9] We adopted the holistic definition of self-management proposed by the US Institute of Medicine.[10]

“Self-management is defined as the tasks that individuals must undertake to live with one or more chronic conditions. These tasks include having the confidence to deal with medical management, role management and emotional management of their conditions.”

**Fig 1:**
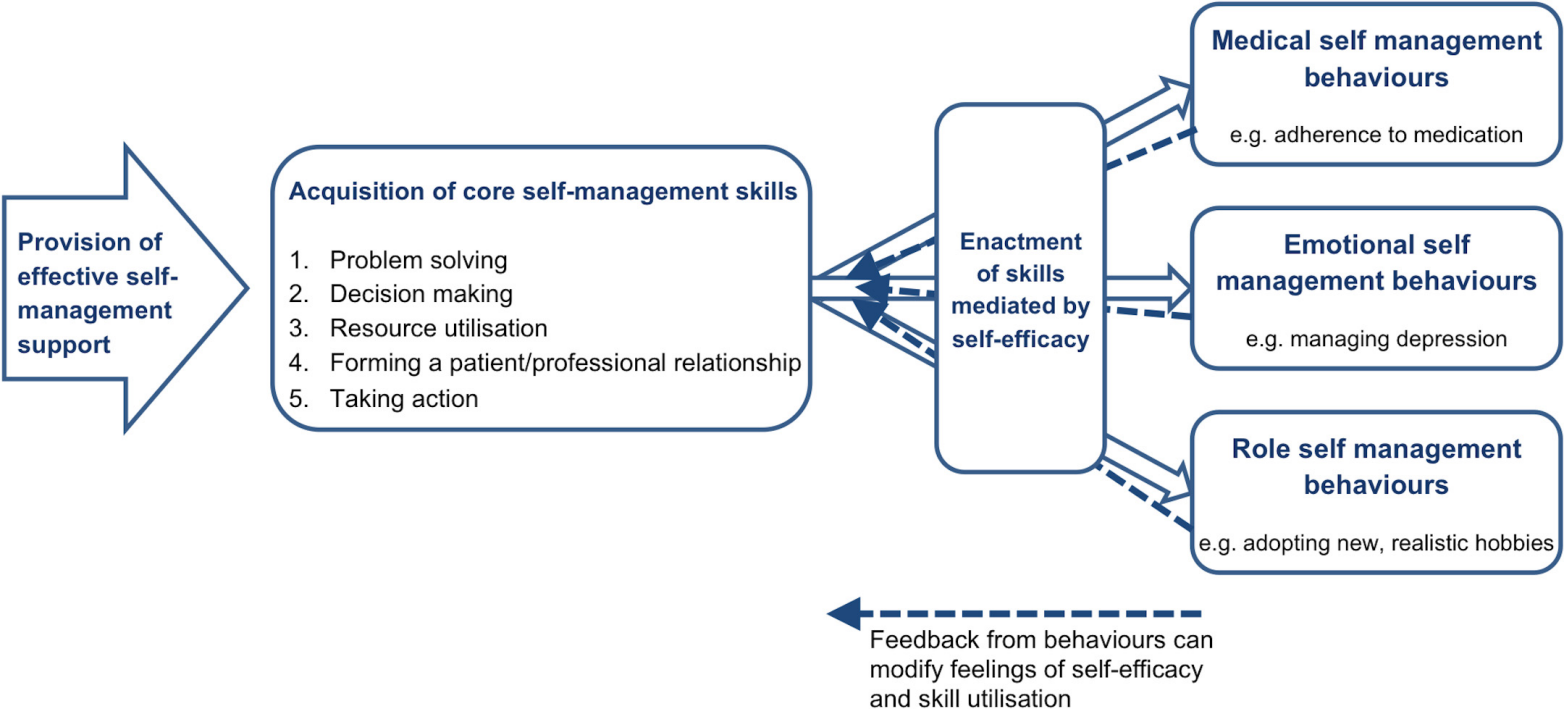
The process of adoption of self-management behaviours.

Medical, role and emotional tasks have been described by Corbin and Strauss as the core components of the management of LTCs.[11] Self-management support in the context of stroke survivorship should therefore aim to empower individuals with the skills to: (1) manage medical tasks (e.g. secondary stroke prevention); (2) maintain or change behaviours or life roles (e.g. dress oneself, return to work); and (3) deal with emotional consequences of stroke survival (e.g. post-stroke depression). To facilitate these, Lorig and Holman identified five core self-management skills: problem solving; decision making; appropriate resource utilisation; forming a partnership with a healthcare provider; and taking necessary actions.[12] Self-efficacy, an individuals’ confidence in their ability to carry out a certain task or behaviour, is commonly viewed as the mediator between the acquisition of self-management skills, and the enactment of self-management behaviours (see Fig 1).[13]

To inform healthcare systems seeking to promote self-management, we performed a meta-review of existing systematic reviews investigating stroke self-management support. The broad perspective that can be achieved by a meta-review makes the outputs particularly relevant for informing policy or clinical practice.[14] This meta-review is part of a systematic overview of the evidence for self-management support of LTCs commissioned by the National Institute for Health Research Health Services and Delivery Research Programme.[15]

## Methods

### Search Strategy and Selection Criteria

Informed by preliminary scoping of the literature, our basic search strategy was; ‘self-management support terms’ AND ‘stroke terms’ AND ‘systematic review terms’. Self-management support search terms included “confidence”, “self-efficacy”, “responsib*”, “autonom*”, “educat*”, “knowledge”, “(peer or patient) ADJ1 (support or group)” and “(lifestyle or occupational) ADJ1 (intervention* or modification* or therapy)” as well as relevant MeSH terms (see Supporting information: S1 Table for full search strategy).

We searched MEDLINE, EMBASE, CINAHL, PsychINFO, AMED, BNI, Cochrane Database of Systematic Reviews, and Database of Abstracts of Reviews for Effectiveness from January 1993 to June 2012. We also hand-searched the journals BioMed Central Systematic Review, Health Education and Behaviour, Health Education Research, Journal of Behavioural Medicine and Patient Education and Counseling. A forward citation search was performed on all included reviews using ISI Proceedings (Web of Science), and all included publication reference lists were screened.

Eligibility criteria were: systematic reviews which searched for randomised controlled trials (RCTs); included individuals with a clinical diagnosis of stroke; reviewed interventions which focused on, or incorporated, strategies to support self-management (as defined above) delivered to stroke survivors, their caregivers, or both; and included outcomes on healthcare service use, health outcomes, health behaviour, quality of life, or self-efficacy of stroke survivors. We excluded: non-English publications; reviews which included a range of study designs or conditions unless they provided separate data for RCTs with stroke survivors; mono-component interventions (e.g if focused on acquiring a specific skill as opposed to broader self-management skills); or if only carer-related outcomes were reported.

Following training to establish consistent practice, the initial screening of titles and abstracts was performed by one reviewer (HLP, EE, or GP) with a 10% check by a second reviewer (HP or ST), with good inter-rater agreement (96%). Full text screening was undertaken by two reviewers (HLP, EE or GP) working independently with 81% agreement; any disagreements were re-screened by the third reviewer, and 10% were checked by a fourth reviewer (HP or ST).

### Quality Appraisal, Data Extraction, Outcomes and Relevance

The quality of all included reviews was appraised using the R-AMSTAR tool,[16] by one reviewer (HLP) with a 10% check by a second reviewer (GP). A review was defined as high quality (score>40), reasonable quality (score 31–39), or low quality (score<30). (See Supporting information: S2 Table for the R-AMSTAR quality criteria).

Data were extracted by one reviewer (HLP) using a piloted data extraction table and the completed tables were checked by a second reviewer (HP) for accuracy with disagreement resolved by discussion.

We extracted the findings and conclusions as synthesised by the authors of the reviews, and specifically avoided going back to the individual RCTs. However, the aims of both the included reviews and the RCTs they included did not always completely match the aims of our meta-review. We therefore assessed the potential relevance of the individual RCTs to our aim and used this, in combination with the quality assessment results, to guide the weight we attached to the conclusions of each review.

Primary outcomes of interest were those we anticipated might benefit most from a self-management intervention: (1) activities of daily living (ADL); (2) extended activities of daily living (extended ADL); (3) self-efficacy; (4) community reintegration, ability to participate in work, leisure or social activities; and (5) quality of life (QOL). Secondary outcomes were; cognitive function, mood, compliance, use of care services, and poor outcome(s) or death. See Table 1 for outcome measure definitions.

**Table 1:** Outcome measure definitions.

Outcomes	Definition	Measures reported in reviews
**Primary outcomes**		
**Primary activities of daily living**	Typically limited to functional ability and personal care (e.g. feeding, bathing and dressing measures)	Barthel index or alternative global dependency scale
**Extended activities of daily living**	Encompasses more complex tasks necessary for community and domestic participation (e.g. shopping, cooking and transportation use)	Frenchay Activities Index, Nottingham Extended ADL, Lawton Independent ADL scale, other unspecified EXTENDED ADL scales
**Self-efficacy**	The confidence that an individual has in their own ability to perform a specific task or behaviour	Recovery efficacy (REFFI), Self-efficacy to perform, Self-efficacy scale
**Community reintegration**	The ability of individuals to reintegrate into their society, including participation in leisure or social activities or work, where relevant	Patient Personal Adjustment and Role Skills measure, Nottingham Leisure Questionnaire, London Handicap Scale, activity limitation
**Quality of life**	Quality of life or subjective health status	Dartmouth Coop Chart, Nottingham Health Profile, the Sickness Index Profile
**Secondary outcomes**		
**Cognitive function**	Functioning in cognitive areas including problem solving, attention, memory, orientation and executive function	CFQ64 cognitive failures in daily life, category test for problem solving, various (unspecified) measures
**Mood**	Anxiety, depression or general mood	Hospital Anxiety and Depression Scale, Beck Depression Inventory, General Health Questionnaire
**Service use**	Use of health care services	Hospital admissions, service contacts or health professional contacts, cost to health and social services
**Compliance**	Modification of health behaviours, risk reduction and performance of required tasks	Millers health behaviour scale
**Poor outcome(s) or death**	Deterioration in ADL, a label of dependency (above or below a defined cut-off point on an ADL scale), requiring institutional care or death	ADL measures as above, dichotomous institutional care measure, or death

### Data Synthesis

Based on our preliminary scoping work, we expected substantial heterogeneity amongst included reviews, several of which would themselves include a heterogeneous group of RCTs. We therefore planned to undertake a narrative synthesis. Interpretation of results was facilitated by discussion amongst the multidisciplinary study team and an end-of-project national workshop.

## Results

**Fig 2:**
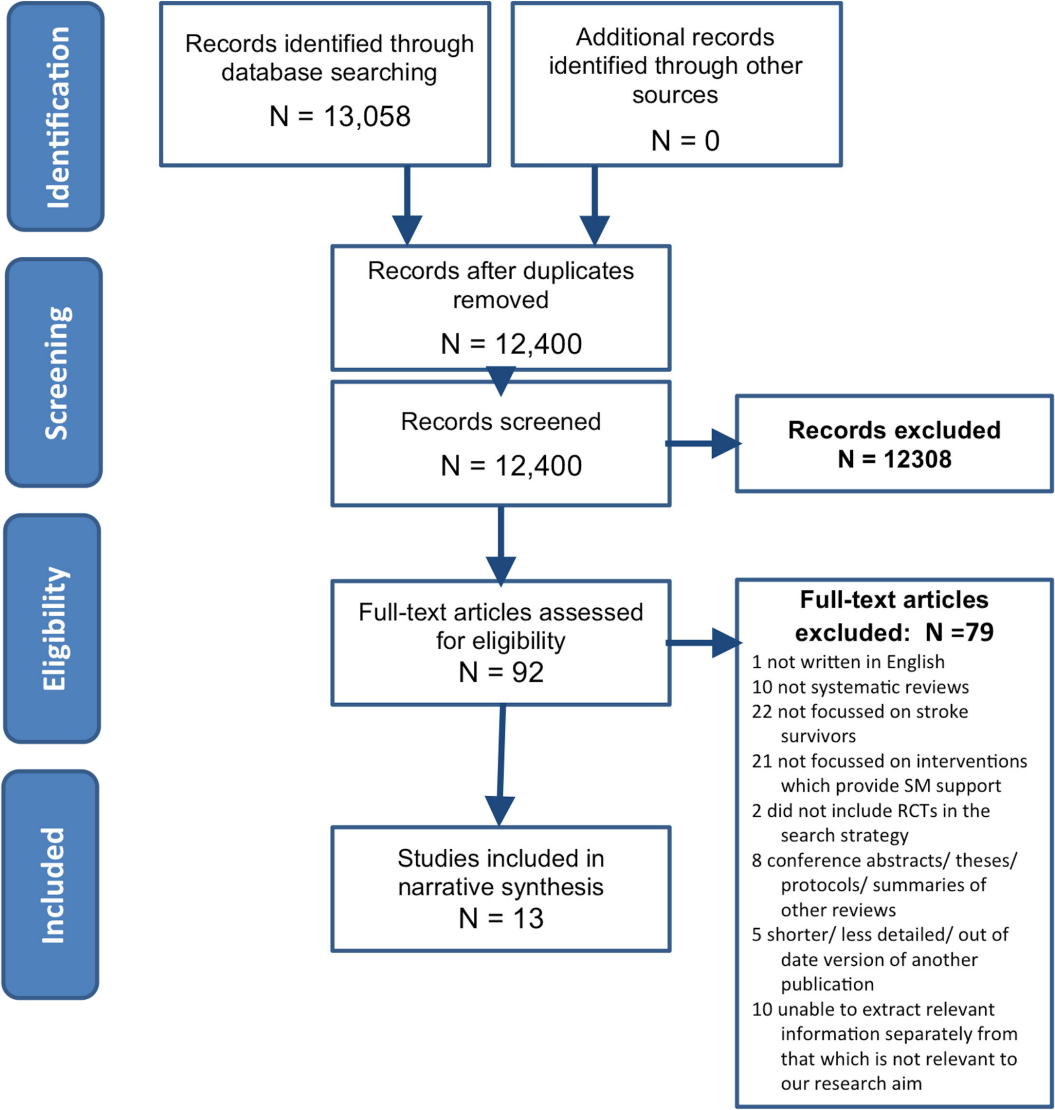
PRISMA flow chart.

Of 12,400 titles and abstracts, 13 reviews were identified for inclusion in our meta-review of self-management support interventions for stroke survivors.[17–29] Fig 2 is the PRISMA flow chart. These reviews collectively represented 101 individual RCTs, 29 of which were included in more than one review (see Supporting information: S3 Table for details of overlapping RCTs). Year of review publication ranged from 2003 to 2012, whilst the year of publication of RCTs included within these reviews dated back to 1981. Specified locations included: UK; USA; China; Australia; the Netherlands; Sweden and Denmark.

### Interventions Identified

Although the term ‘self-management’ was rarely used, by reference to our definition and the underlying theoretical basis for self-management,[12,13] we identified interventions which provided components of self-management support. Table 2 summarises the characteristics of the RCT interventions included in each review and an explanation of why we considered that these interventions represented components of self-management support. See Supporting information: S4 Table for further detail.

**Table 2:** Characteristics of the RCT interventions included in the systematic reviews.

Review	Review aim(s)	Inclusion criteria for interventions	Why this is SM support	Setting	Components included	Timing	Duration, intensity
**Therapy rehabilitation**							
**Aziz 2008 [17]**	Do therapy-based rehabilitation services influence stroke survivor outcomes a year or more after the index stroke?	Outpatient based rehabilitation, provided by physiotherapist, occupational therapist or multidisciplinary staff, working with patients to improve task-orientated behaviour. The intervention must require an organisational and staffing structure, and must be delivered 1 year post stroke.	All trials showed an approach based on problem solving, aiming to reduce disability by altering task-orientated behaviour and goal-orientated activities.	Patients’ home or in outpatient rehabilitation centres. Part of therapists’ usual work. Intervention performed by existing community physiotherapy service.	Approaches adopted by trials were single or multidisciplinary interventions, some using problem solving approaches.	1 year post stroke.	Fixed or flexible regimes. Programme duration ranged from 12 weeks to a year, and varied in their intensity.
**Hoffman 2010 [18]**	Is OT for people with cognitive impairment post- stroke effective in improving functional/cognitive abilities?	OT interventions for cognitive impairment in people with stroke offered three approaches. Remedial approach: training specific cognitive deficits. Compensatory approach: training skills for daily activities, use of assistive devices, educating patients/caregivers about strategies to compensate for cognitive impairment. Dynamic interactional approach: integrating remedial and compensatory elements.	Training advised and educated strategies to overcome patients’ cognitive impairment.	Inpatients. Delivered on an individual basis.	Cognitive skills remediation training.	Hospital based following acute stroke.	Training administered 30–40 minutes 3 times a week for an average of three to four weeks.
**Legg 2006 [19]**	Do interventions provided by OTs, which aim to facilitate personal ADL, improve outcomes for stroke survivors?	OT interventions which either focussed on practice of personal activities of daily living or were targeted towards improving the patient’s ability to perform personal activities of daily living. Occupational therapists working as part of a multidisciplinary team were excluded.	Aims to enable people to achieve health, well-being and life satisfaction. Promotes recovery through the use of purposeful activities.Targets patient’s ability to perform ADL.	Home based. Delivery on an individual basis	Range of OT interventions including: OT based on leisure activities or activities of daily living; teaching new skills; use of adaptive equipment; carer involvement; goal setting; information provision; liaison with other services; facilitating return of function.	Mainly following admission to or discharge from inpatient facilities.	Programmes of between 6 weeks and 6 months. Number of visits ranged from approximately 2·5 to 18.
**OST 2003 [20]**	Do therapy-based rehabilitation services influence stroke survivor outcomes?	Therapy based rehabilitation service interventions delivered to stroke patients resident in the community. Interventions must be provided or supervised by qualified physiotherapy, occupational therapy or multidisciplinary staff, who work with the patient to improve task-orientated behaviour primarily aiming to reduce disability.	Included problem solving and education aimed at reducing disability.	Mostly home based, some delivered in rehabilitation centres. Delivery mainly on an individual basis, face to face.	PT, OT or MDT input. Components including: teaching skills; facilitating return of function; information provision; equipment; adaptations; advice on financial assistance and transport; liaison with specialists; managing psychosocial stressors.	Various. Mainly at discharge from inpatient facilities.	Duration ranged from 5 weeks to 6 months. Intensity ranged from daily visits, to an average of one visit every 8 weeks.
**Poulin 2012 [21]**	Do executive function interventions improve executive functions and functional abilities in daily life?	Cognitive interventions to remediate executive function impairments or improve functional tasks compromised by impairments in executive function (excluding attentional processes). Components such as computerised cognitive training, problem solving, and strategy formation techniques, goal management training, or other compensatory strategies and external aids for overcoming everyday executive problems were all considered.	Involved components such as problem solving, strategy formation techniques, goal management training, and other compensatory strategies and external aids for overcoming everyday executive problems.	All interventions were delivered remotely to individuals in a home based setting except in one sub-group where strategy training was delivered face-to-face by therapists.	Heterogeneous interventions. Working memory: computerized tasks, auditory and visio-spatial stimuli. Strategy training: problem solving, planning, multitasking, and goal management. External compensation: electronic prompts to carry out tasks e.g. taking medication, appointments.	Chronic (> 6 months post stroke).	Duration between 5 and 20 weeks. Sessions (where applicable) lasted 40–45 minutes, and occurred between 1 and 5 times a week.
**Steultjens 2003 [22]**	Do OT interventions improve outcomes for stroke survivors?	OT interventions in 6 categories: (1) training of sensory-motor functions; (2) training of cognitive functions; (3) training of skills such as dressing, performing domestic activities; (4) instruction in the use of assistive devices; (5) provision of splints and slings; and (6) education of family and caregivers. Comprehensive OT included all 6 categories.	Interventions aimed to facilitate task performance by improving skills or developing compensatory strategies to overcome lost skills. Included advice, education.	Often unclear, but majority were home based and delivered on an individual basis, others were delivered in an inpatient setting.	Components included; client centred OT; enhanced OT; teaching new skills; facilitating ADL and return of function; enabling use of equipment; counselling of patient and caretaker; intellectual training; and strategy training.	Often unclear, but generally less than 1 year since stroke.	Sessions of 30 to 52 minutes occurring once or twice a week over 6 weeks and 6 months.
**Walker 2004 [23]**	What is the efficacy of community OT?	Home-based OT in patients with a clinical diagnosis of stroke. 2 approaches to the OT intervention are defined. (1) ADL interventions encouraging patients to participate in personal and extended activities of daily living; (2) leisure therapy interventions aiming to improve leisure participation.	Primarily concerned with the re-ablement and re-settlement of individuals into their chosen home environment.	Delivered by research occupational therapists or clinicians in a home based setting (including care or nursing homes).	Components included; training in activities of daily living; leisure therapy; and both.	Not reported.	Between 5 and 10+ sessions delivered over 6 weeks to 6 months.
**Other SM support**							
**Ellis 2010 [24]**	What is the efficacy of stroke liaison workers in increasing participation and improving wellbeing of stroke survivors?	Referral to a stroke liaison worker who provided a multifaceted service including: education and information provision, social support and liaison with other services. Often provided from the point of patient discharge from hospital. Studies were excluded where the intervention was judged to be single-faceted.	Aim to increase participation and improve wellbeing for patients and carers. Typically provide emotional and social support and information.	Mostly with urban populations. Delivery home based; face to face or via telephone.	Interventions were either proactive or reactive, and adopted either a structured, flexible, or focussed approach.	Various. Mainly 2–6 weeks since stroke onset.	Between 3 and 15 contacts, each lasting 15–90 mins over a maximum of 9 months.
**Ko 2010 [25]**	Do patient-held medical records improve clinical care, patient outcomes or satisfaction?	The patient holds a copy of the paper-based medical record, take to health appointments, help manage healthcare tasks and communication. May be with or without other interventions such as additional education for staff, reminder posters in clinics, and/or dedicated patient held record coordinating staff. The review excluded electronic health records, including those controlled by the patient.	Aimed to manage healthcare tasks/communication, to enable continuity and quality of care. Records included key patient and healthcare information, and space for patient note-taking.	No RCTs identified in stroke survivors.	N/A	N/A	N/A
**Korpershoek 2011 [26]**	What self-efficacy enhancing interventions influence mobility, ADL, depression and HRQL?	Self-efficacy enhancing interventions for stroke patients. Interventions must aim to increase confidence in one’s ability to perform a task or specific behaviour. Interventions must also be feasible and suitable to be delivered in nursing practice.	Self-efficacy is the confidence in one’s ability to perform a task or specific behaviour. A high sense of self-efficacy leads to desired outcomes.	Community or hospital rehabilitation settings.	A heterogeneous group of interventions to enhance self-efficacy: psychosocial intervention; computer-generated tailored written information; the Chronic Disease Self-Management Course education; task-oriented walking intervention.	Various. Ranged from acute to within 1 year of stroke onset.	Insufficient detail. One intervention was delivered three times a week for 6 weeks.
**Lui 2005 [27]**	Is teaching problem solving skills to caregivers in stroke care effective?	Educational interventions for problem solving delivered to family caregivers in stroke care. Interventions involve teaching family caregivers to cope with problems and to relieve stress.	Teaching family caregivers to cope with problems. Problem solving strategies included positive problem orientation and goal setting.	Delivery: class training, home visits, or telephone contact. (Most was provided in their home by healthcare professionals)	Problem solving strategies taught included: positive problem orientation; confronting the problem; analysing the problem; and goal setting.	Mostly applied in the early post-stroke period.	Duration ranged from 2 to 12 months. On average, each home visit lasted 1 to 2 hours.
**Rae-Grant 2011 [28]**	What is the efficacy of self-management in people with chronic neurological conditions?	Self-management interventions for neurologic disorders. Interventions collaboratively help patients and families acquire skills and confidence to manage their illness, providing self-management tools, and routinely assessing problems and accomplishments.	Helping patients and families acquire the skills and confidence to manage their illness, by providing self-management training.	No RCTs identified in stroke survivors.	N/A	N/A	N/A
**Smith 2008 [29]**	What is the effectiveness of information strategies provided with the intention of improving outcomes for stroke survivors or their caregivers?	Information intervention delivered to stroke patients, and/or their caregivers with the intention of improving outcomes. Information may be active (following information provision there was purposeful attempt to allow participants to assimilate information and subsequently clarify/consolidate) or passive (single occasion of information provision with no follow up or consolidation). Trials were excluded in which information giving was only one component of a more complex rehabilitation intervention.	Information strategies provided with the intention of improving the outcome for stroke patients or their identified caregivers or both.	Delivery setting varied and included home based, outpatient, inpatient and rehabilitation units.	Active interventions included; programmes of lectures; opportunities to ask questions or to contact specialist nurses for further information; hands on training; phone calls; interactive workbooks; regular reviews; personalised records detailing risk factors and targets; counselling. Passive interventions included; written information sometimes tailored to the individual.	Prior to discharge in 8 trials. Between 1 and 24 months post discharge in the remaining 9.	Between 1 and 8 contacts lasting between 30 minutes and 2 hours each. Intervention length varied from a one-off to 6 months In some studies there was no contact).

Seven reviews explored interventions based on therapy rehabilitation,[17–23] though the focus of the interventions varied. Hoffman 2010, and Poulin 2012, looked at interventions designed specifically for people with cognitive impairment.[18,21] The remaining reviews explored therapy rehabilitation generally,[17,20] or occupational therapy (OT) specifically.[19,22,23] Self-management components in the therapy-based interventions included: problem solving; remediation training; goal setting; information provision; support with adaptive equipment; liaison with other services; and training in ADL. The majority of interventions were home-based and delivered to individuals on a face-to-face basis, though other models included delivery in an outpatient rehabilitation centre, or group setting. Delivery of the therapy rehabilitation was initiated soon after the acute stroke event in five reviews,[18–20,22,23] and later in stroke recovery (six months to more than one year) in two reviews.[17,21] Outcomes were measured between one week and 12 months after the end of the intervention period.

The remaining six reviews looked at various self-management support interventions including referral to stroke liaison workers,[24] information provision,[29] self-efficacy enhancement,[26] patient held records,[25] and caregiver problem solving.[27] Rae-Grant 2011 was the only review that explicitly examined self-management programmes.[28]

### Quality and Relevance Assessment

Table 3 (with further detail in Supporting information: S5 Table) gives the results of the R-AMSTAR quality assessment and the judgements made on the relevance of the individual RCTs included within the reviews. R-AMSTAR scores ranged from 24 to 42 out of a possible total of 44. In seven reviews,[17,19,20,23,24,27,28] the majority of RCTs were deemed to be self-management interventions and so the review findings were judged to be highly relevant to our review aim.

**Table 3:** Relevance and quality of systematic reviews.

	Primary study designs identified to answer relevant review question	Total number of RCTs extracted	Number of extracted RCTs judged to include SM support	R-AMSTAR total score /44
**Therapy rehabilitation**				
**Aziz, 2008 [17]**	RCTs	5	5	40
**Hoffman, 2010 [18]**	RCTs	1	0	35
**Legg, 2006 [19]**	RCTs	9	8	42
**OST, 2003 [20]**	RCTs	14	11	41
**Poulin, 2012 [21]**	Controlled and uncontrolled designs	3	1	32
**Steultjens, 2003 [22]**	Controlled and uncontrolled designs	18	6	32
**Walker, 2004 [23]**	RCTs	8	8	35
**Other SM Support**				
**Ellis, 2010 [24]**	RCTs	16	16	35
**Ko, 2010 [25]**	None identified	0	0	31
**Korpershoek, 2011[26]**	RCTs	4	2	24
**Lui, 2005 [27]**	Quantitative and qualitative designs	6	6	24
**Rae-Grant, 2011[28]**	None identified	0	0	27
**Smith, 2008 [29]**	RCTs	17	9	40

Relevance of the interventions reported in the RCTs included in the systematic reviews was assessed on the basis of the detail provided in the review report. The quality of reporting details about the interventions varied between the reviews so that some judgement was required.

### Intervention Results

Table 4 documents the findings of each review and our interpretation of these results.

**Table 4:** Findings of the systematic reviews.

Review Intervention focus	n. RCTs included (n. relevant)	Total n. participants	R-AMSTAR quality rating/44	Time at which outcomes measured	Primary and Secondary Outcomes Beneficial effect +Harmful effect –No significant effect 0	Narrative synthesis	Meta-analysis	Significant findings	Interpretation
**Therapy rehabilitation**									
**Aziz, 2008 [17]** Rehabilitation 1 year post stroke	5 (5)	487	40	3–12 months	**1**° ADL / Extended ADL		0/0		Inconclusive whether intervention was able to influence any other relevant patient outcome one year after stroke.
					QoL		0		
					**2**° Mood		0		
					Poor outcome(s) or death		+	Difference in poor outcome or death (51% versus 76%) (95% CI 3% to 48%; P = 0·03).	The only positive finding is based on a single study.
**Hoffman, 2010 [18]** OT for cognitive impaired	1 (0)	33	35	NS	**1** ^**o**^ ADL	0		No significant findings to report.	There is a paucity of RCTs evaluating cognitive rehabilitation in stroke survivors as only 1 RCT was identified.
**Legg, 2006 [19]** OT rehabilitation	9 (8)	1258	42	3–12 months	**1** ^**o**^ ADL		++	Improved ADL (SMD 0·18; 95% CI 0·04 to 0·32; P = 0.01)	OT rehabilitation has positive outcomes on personal activities of daily living.
					Extended ADL		+	Improved extended ADL (SMD 0·21; 95% CI 0·03 to 0·39; P = 0·02).	
					QoL		0		
					**2** ^**o**^ Mood		0		
					Poor outcome(s) or death		+	Reduction in odds of a poor outcome or death (OR 0·67; 95% CI 0·51 to 0·87; P = 0·003). Reduction in odds of deterioration or death (OR 0·60; 95% CI 0·39 to 0·91; P = 0·02).	
**OST, 2003 [20]** Therapy rehabilitation	14 (11)	1617	41	3–12 months	**1** ^**o**^ ADL		+	Increased ADL scores (SMD 0·14, 95% CI 0·02 to 0·25; P = 0·02).	Both positive outcomes indicate therapy based rehabilitation to have a positive effect on personal activities of daily living.
					Extended ADL		++	Increased extended ADL scores (SMD 0·17, 95% CI 0·04 to 0·30; P = 0·01).	
					QoL		0		
					**2** ^**o**^ Mood / Service use		0/0		
					Poor outcome(s) or death		++	Reduction in the odds of a poor outcome or death (OR 0·72; 95% CI 0·57 to 0·92; P = 0·009).	
**Poulin, 2012 [21]** Therapy rehabilitation for cognitive impairment	3 (1)	109	32	NS	**2** ^**o**^ **Working memory training** Cognitive function	++		**Working memory training sub-group (chronic)** Reduction in cognitive failures (effect size = 0·80; P = 0·005).	All findings are based on a single study so are taken with caution.
					**1** ^**o**^ **Strategy training** Extended ADL	++		**Strategy training sub-group (chronic).** Positive effects on extended ADL (P <·01). Improvement in problem solving self-efficacy was greater for face to- face group compared to self-paced computer assisted training, or online though video conferencing (F = 6·45; P = 0·003).	Strategy training is the only intervention which meets our definition of SM support. The review offers some support for the effectiveness of strategy training on improving extended activities of daily living.
					**2** ^**o**^ **External compensation** Compliance	++		**External compensation sub-group (chronic)** Improved compliance in activities (z = 2·953, P = 0·003)	All RCTs involved individuals in the chronic phase of recovery, highlighting need for research into cognitive rehabilitation at early stages.
**Steultjens, 2003 [22]** OT rehabilitation	18 (6)	1825	32	NS	**Comprehensive OT**				Comprehensive OT was found to positively affect more outcomes than any of the other sub-groups, and is the only sub-group which meets our SM support definition. The outcomes reported for comprehensive OT are a composite of 6 RCTs.
					**1**° ADL / Extended ADL		+/0	Small but significant effect sizes on ADL (SMD 0·31; 95% CI 0·03 to 0·60).	
					Community reintegration		0		
					**Cognitive function**				
					**1** ^**o**^ ADL		0		
					**Training of skills**				Isolated OT elements were found to be much less effective than comprehensive OT; only skills training found any beneficial effects and these were based on a single study so must be taken with caution.
					**1** ^**o**^ ADL		+*	Significant effect on ADL in one study (SMD 0·46; 95% CI 0·05 to 0·87)	
					Extended ADL		+*	Significant effect on extended ADL in another study (SMD 2·29; CI 1·26 to 3·32)	
					**Cognitive vs training of skills.**				
					**1**° ADL / Extended ADL		0/0		
					**2**° Cognitive function		0		
					**Advice about assistive devices.**				
					**1**° QoL		0		
									No RCTs were found exploring education of family or caregivers by an OT. Whilst education provision is an important role of an OT, it is something that is unlikely to be done in isolation, this may explain the paucity of RCTs in this area.
**Walker, 2004 [23]** OT rehabilitation	8 (8)	1143	35	End of intervention 1.25–6 months. End of trial 4.5–12 months.	**1**° ADL		+*	OR 0·71; 95% CI 0·52 to 0·98	The duration/intensity of intervention did not mediate the effect on the primary outcome. This review supports OT rehab, demonstrating positive effects on extended ADL and leisure scores.
					Extended ADL		+*	WMD 1·30 points; 95% CI 0·47 to 2·13	The effect on extended ADL varied by age; older patients appeared to benefit more than younger ones (P = 0·01).
					Community reintegration		+*	WMD 1·51 points; 95% CI 0·24 to 2·79	
					**2**° Mood		0		
					Poor outcome(s) or death		0		
					**OT emphasising ADL**				
					**1**° Extended ADL		+*	WMD 1·61 points; 95% CI 0·72 to 2·49	
					Community reintegration		0		
					**OT emphasising leisure**				Patients with lower levels of dependency appeared to benefit more in leisure scores (WMD 2·86 points; 95% CI 0·70 to 5·02).
					**1**° Extended ADL		0		
					Community reintegration		+*	WMD 1·96 points; 95% CI 0·27 to 3·66	
**Other SM support**									
**Ellis, 2010 [24]** Stroke liaison	16 (16)	4759	35	NS	**1**° ADL		0		No positive overall effects were demonstrated for stroke liaison.
					Extended ADL		0		
					Community reintegration		0		
					QoL		0		
					**2**° Mood		0		
					Poor outcome(s) or death		0		
					**Education and information**				
					**1**° QoL		+	SMD -0·24; 95% CI -0·44 to -0·04; P = 0·02	
					**Barthel 15–19**			**(mild to moderate disability)**	
					**2**° Poor outcome(s) or death		+	Significant reduction in dependence (OR 0·62; 95% CI 0·44 to 0·87; P = 0·006), and death or dependence (OR 0·55; 95% CI 0·38 to 0·81; P = 0·002).Significant subgroup heterogeneity found for the Barthel 15–19 group (Chi2 P < 0·05).	Post-hoc analysis found positive effects for those individuals with mild to moderate disability
**Ko, 2010 [25]** Patient held medical records	0 (0)	0	31	Found no RCTs – no outcomes to report.	Found no RCTs – no outcomes to report.			Found no RCTs – no outcomes to report.	No RCTs were identified which studied the use of patient held medical records in stroke survivors. This highlights an area of potential stroke SM where more primary research is required.
**Korpershoek,2011 [26]** Self-efficacy enhancing	4 (2)	630	24	6 and 12 month (Chronic disease SM), NS/NR for others.	***Chronic Disease SM Course***				Only the chronic disease self-management course definitely met our definition of SM support and that showed positive results on a range on health-related quality of life outcomes. However, the results from this review must be taken with caution as each sub-group represents a single study.
					**1**° Self-efficacy	0			
					Community reintegration	0			
					QoL	++		Significant positive effect on HRQL outcomes including mobility (P < 0.01), self-care (P < 0·001), thinking (P < 0·01), and social roles (P < 0·001). ***Computer-generated tailored information sub-groupm*** Anxiety scores changed significantly in favour of control, (95% CI 0·2 to 2·8, P = 0·03).	
					**2**° Mood	0			
**Lui, 2005 [27]** Caregiver problem solving	6 (6)	1676	24	2 weeks- 12 months	**1**° ADL	0			
					Self-efficacy	0			
					Community reintegration	+		Better patient adjustment at 12 months after stroke (P<0·01). Improvement of social outcome in patients with mild disability at 6 months (P = 0·03).	The reported positive results represent only 1 study each. (Only 3 of 6 RCTs reported outcomes for stroke survivors).
					**2**° Mood	0			
**Rae-Grant, 2011 [28]** SM	0 (0)	0	27	Found no RCTs	Found no RCTs – no outcomes to report.			Found no RCTs – no outcomes to report.	There is an absence of RCTs explicitly investigating stroke self-management.
**Smith, 2008 [29]** Information provision	17 (9)	2831	40	1 week-1 year	1° ADL		0	.	We take active, but not passive, information provision to be SM support.
					Community reintegration		0		
					QoL		0		
					2° Mood		++	Clinically small benefit of information provision on depression (WMD -0·52; 95% CI, 0·93 to -0·10; P = 0·01) Active information provision significantly more effective than passive information for depression (P < 0·02 for all the trials), and anxiety (P < 0·05 for trials reporting dichotomous data, P < 0·01 for trials reporting continuous data)	This review provides evidence that active information has a positive impact on anxiety and depression in stroke survivors
					Service use / Compliance		0/0		
					Poor outcome(s) or death		0		

0 No evidence of effect (P> 0·05) + Some evidence of effect in favour of intervention/control (0·05 ≥P> 0·01) ++ Strong evidence of effect in favour of intervention/control (0·01≥P> 0·001)

* No p values provided, there is *at least* some evidence of effect, but may underestimate true effect size

The only review that searched for interventions described as self-management,[28] did not identify any RCTs delivered to stroke survivors, suggesting that there is a paucity of evidence exploring the concept of ‘self-management’ within stroke care.

The interventions described in the different reviews were diverse but six could be grouped as therapy-based interventions. We present a synthesis of our findings below, considering first the primary and then secondary outcomes and (where relevant) sub-group analyses.

### Therapy Rehabilitation: Primary Outcomes

The primary outcome of ADL was assessed in six reviews of therapy rehabilitation, with four of these reviews overlapping substantially in the RCTs included.[19,20,22,23] Two high quality, highly relevant reviews,[19,20] and two reviews of reasonable quality,[22,23] reported some evidence,[20,22,23] or strong evidence,[19] of beneficial effect on ADL five weeks to 12 months after intervention delivery. One review of reasonable quality which had an overlap of just one RCT found no effect on ADL.[18] The only review to search for therapy rehabilitation delivered one year post-stroke found no beneficial effect on ADL.[17]

Outcomes for extended ADL were also reported in six reviews,[17,19–23] with three identifying some,[19,22,23] two finding strong,[20,21] and one review finding no evidence of benefit.[17] Of these, two looked exclusively at interventions delivered in the late phase of stroke recovery; one finding no benefit,[17] and the other (based on a single study) finding strong evidence of benefit.[21]

Two reviews reported measures of community reintegration,[22,23] both of reasonable quality, and both identified a significant trend favouring therapy intervention.

The three highest quality reviews, all of high relevance, reported QOL outcomes, however none demonstrated any significant benefit.[17,19,20] Mood was assessed in four reviews, including the three highest quality reviews, with no significant benefits reported.[17,19,20,23] One high quality review assessed service use and found no intervention effects.[20] Compliance was reported in one review of reasonable quality which found a significant, positive effect in one RCT.[21] Cognitive function was reported in two lower quality reviews, both of low relevance, with one finding positive effects in one RCT,[21] and the other finding no effect.[22]

### Therapy Rehabilitation: Secondary Outcomes

The composite measure of poor outcome (deterioration in ADL, dependence/institutional care or death) was reported in the three highest quality reviews, all finding significant beneficial effects.[17,19,20]

### Other Models of Self-Management Support

A high quality review of interactive information provision (see Table 2 for specific examples) found strong evidence of a beneficial impact on mood, though the effect was small and of doubtful clinical significance.[29] A lower quality review of interventions to enhance self-efficacy found that a chronic disease self-management course had a significant positive effect on QOL.[26] Based on only one RCT, a lower quality review exploring problem solving delivered to caregivers identified positive influences on community reintegration.[27] The remaining two reviews identified no RCTs of stroke survivors.[25,28]

The review of stroke liaison workers, whilst finding no overall benefit in subjective health status, identified a significant effect on QOL for the sub-group of interventions with an emphasis on education and information provision.[24]

### Sub-Group Results

Sub-groups of therapy-based interventions that appeared to have most impact on primary outcomes included comprehensive occupational therapy (as opposed to specific skills training) on ADL,[22] and face-to-face training groups (as compared to video conferenced or computer-based interventions) on problem solving self-efficacy.[21] Targeted interventions were associated with significant increases in the outcome of primary focus, but tended not to be associated with benefits in other domains.[23] These sub-group results are from reviews of reasonable quality.

Walker’s 2004 high quality review of therapy rehabilitation found that effects varied by age; older patients appeared to gain more benefit in extended ADL skills than those who were younger.[23] Those with the most severe disability were found to gain least from the support interventions: stroke liaison workers reduced dependence in individuals with mild to moderate, but not severe disability.[24] Therapy rehabilitation achieved a non-significant improvement in community reintegration for patients with lower levels of dependency.[23]

## Discussion

### Summary of Principal Findings

We found little evidence specifically using the terminology ‘self-management’ in the stroke literature. However, core elements of self-management support including problem solving, decision making, and goal setting are delivered to stroke survivors and their caregivers within the context of therapy rehabilitation. High quality evidence demonstrates that therapy rehabilitation incorporating these elements delivered soon after a stroke improves ADL and extended ADL and reduces the risk of ‘poor outcome’. There is some evidence that early rehabilitation facilitates reintegration into the community. The limited evidence related to therapy rehabilitation delivered a year or more after the index stroke suggests some benefits on extended ADL and risk of ‘poor outcome’.

The reviews exploring other forms of self-management support found evidence to suggest that active information provision has a small, beneficial effect on mood, educational support from stroke liaison services can improve QOL, and caregiver problem solving facilitated community reintegration.

**Fig 3:**
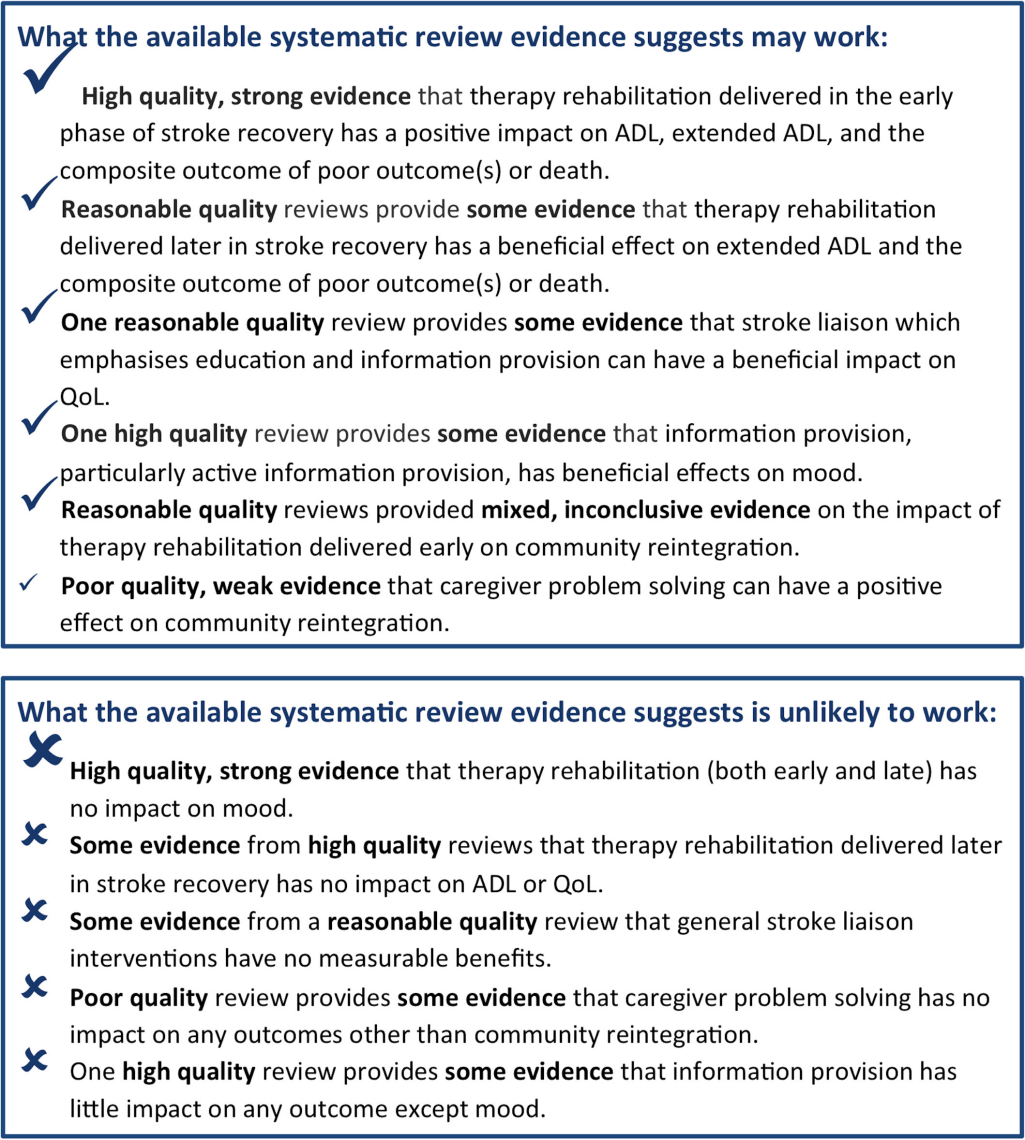
Summary of what the evidence shows.

The strength of evidence for these findings is summarised in Fig 3.

### Strengths and Weaknesses of the Study

In addition to adhering to recommended systematic review search strategies, a strength of our methodology was the regular meetings between team members, whose multidisciplinary backgrounds encompassed public health, primary care and health psychology, enabling a balanced interpretation.

By undertaking a meta-review we were able to synthesise the evidence relating to a broad range of different approaches to addressing our topic of interest, thus providing a convenient overview for policy makers, commissioners of healthcare services and clinicians to inform decisions on the provision of supported self-management for people living with the effects of a stroke.[30] However, meta-reviews of systematic reviews have some intrinsic limitations. We were reliant upon the review authors providing accurate and detailed descriptions of RCTs, and re-synthesis of materials already synthesised risks further loss of detail. To address these issues we appraised the quality of all reviews using R-AMSTAR,[16] and used these scores alongside relevance scores, to inform the weighting of evidence. Additionally, where reviews did not provide adequate narrative descriptions of interventions, we referred to tabulated details in appendices where present.

This meta-review was a commissioned, policy-focused ‘rapid’ review, meaning that screening and data extraction were conducted by one reviewer, and not two reviewers working independently. Whilst we acknowledge this as a potential weakness, we ensured all reviewers were trained before commencing screening, conducted a 10% check of all screening, and report agreement levels. Data extraction forms were also checked by a second reviewer to ensure data integrity.

A widely encountered problem for many review authors was the heterogeneity of RCTs which limited, or prevented, meta-analysis. This also presented challenges for our meta-review. Whilst we planned to conduct a narrative synthesis from the outset, the heterogeneity of the reviews within the ‘other self-management’ category limited the conclusions we could draw. However, we identified seven reviews exploring therapy rehabilitation, providing a more convincing depth of evidence.

Self-management is only one component of therapy, and the benefits we observed may relate to other aspects of the rehabilitation programme. However, in the context of a complex intervention, such as supported self-management, it is rarely possible to isolate the impact of one component from the clinical context. Our inclusion criteria ensured that all the reviews we included explicitly included trials that evaluated aspects of self-management support, and we excluded reviews reporting mono-component interventions focussing on developing a specific task. In addition, many of our outcomes reflected self-management skills such as coping with daily living and reintegration into the community.

We excluded reviews where we were unable to extract RCTs separately from other study designs. This restricted the number of reviews we were able to include, and may have resulted in the omission of important evidence. On the other hand, our strict inclusion criteria ensured that included reviews provided a high level of relevant evidence.

### Evidence for Interventions

#### Therapy rehabilitation supports self-management

In the relatively new and emerging field of stroke self-management, the term ‘self-management’ was poorly recognised and infrequently utilised. As reviewers this challenged us to think reflexively and adaptively about what it really meant to self-manage. Lorig and Holman describe five skills central to self-management. These include supporting the acquisition of problem solving skills, decision making, and taking action (goal setting, or action plans), all prominent features of many stroke rehabilitation programmes.[12] In contrast to action plans in other LTCs which focus on planning for clinical emergencies, for example managing acute asthma,[31] ‘taking action’ in the context of stroke focuses on setting goals towards task accomplishment. The on-going symptoms of stroke survival means self-management must support individuals to cope with and adapt to disability; core aims of therapy provision.

A described element of self-management support is the forming of a patient/healthcare provider partnership.[12] Whilst this was not explicitly described in the reviews of therapy-based rehabilitation, it is a key feature in the work of OTs and other allied therapists, and may therefore be implicit in the therapy-based interventions.[32] The remaining skill described by Lorig and Holman is the ability to find and utilise resources. The provision of such information is a prominent feature of stroke liaison interventions,[24] and has been identified by stroke survivors and their caregivers as a useful service.[33]

The commonalities between stroke rehabilitation programmes and self-management support have also been recognised by Jones, who noted that the aims of rehabilitation often involved increasing problem-solving self-efficacy, constructing action plans, and making decisions, all prominent elements of self-management support.[34] A stated goal of OT is to promote a sense of self-efficacy.[32] Self-efficacy beliefs are an acknowledged mediator of self-management,[19] further supporting a significant role for OT in supporting self-management. Whilst our meta-review demonstrates the specific value of therapists in the context of stroke, effective implementation of self-management requires a whole systems approach in which an integrated healthcare organisation actively promotes collaborative/communicative relationships between enabled patients and motivated healthcare professionals.[35]

#### Medical, role and emotional management

Our definition of self-management support encompasses medical, role and emotional management.[10] The main beneficial effects identified in this review (ADL, extended ADL and ‘poor outcome’) reflect the needs of stroke survivors in the early phase of adjustment. Our parallel synthesis of the qualitative evidence on the experiences of stroke survivors highlights the long-term and frustrating process of adjustment after stroke, and the consequent feelings of increasing social isolation.[15] The early focus mediated by rehabilitation therapists on basic function-related goals needs to merge into later interventions which support reintegration into society through supporting the adoption of more meaningful societal roles. Our data provide less clear evidence as to what format this late phase support should take, though some positive effects on community reintegration and mood were identified.[22,23,27]

Emotional tasks involve being able to deal with psychological responses such as post-stroke depression; only one review found a (clinically small) significant benefit on mood.[29] Current (often therapy-based) interventions are not providing adequate support to enable individuals to self-manage emotional tasks, and future interventions should address this gap.

Medical tasks were rarely explored in the included reviews, but such tasks provide the foundation of secondary stroke prevention and modification of risk factors is an important element of self-management. Lawrence and colleagues found lifestyle interventions such as diet modification and smoking cessation could affect positive behavioural change in stroke survivors;[36] more explicit support to enable individuals to adopt such behaviours should therefore be considered in future self-management support interventions.

## Conclusions and Implications

In contrast to conditions such as asthma and diabetes in which the concept of self-management has been widely explored, evaluated and recommended by guidelines,[31,37] self-management terminology is rarely used in the context of stroke. However, therapy rehabilitation currently successfully delivers elements of self-management support to stroke survivors and their caregivers.

UK national clinical stroke guidelines now recommend offering all patients training in self-management skills, acknowledging the benefits to be gained by providing such support.[38] Those developing stroke self-management support interventions should recognise and respond to the changing needs of stroke survivors as they progress from the acute stroke event through early rehabilitation to long term survivorship. This should include supporting self-management of more complex social roles as well as empowering stroke survivors to manage emotional and medical tasks. Research is needed to explore a new model of stroke self-management which is integrated across secondary, primary, and community care and adopts a whole systems perspective.

## Supporting Information

S1 Table Full Medline search strategy. (DOCX)

S2 Table R-AMSTAR criteria for quality assessment. (DOCX)

S3 Table Number of overlapping RCTs between systematic reviews. (DOCX)

S4 Table Why the included systematic reviews are self-management support. (DOCX)

S5 Table Detailed R-AMSTAR results. (DOCX)
