# Supplementary material for: Self-Management Support Interventions for Stroke Survivors: A Systematic Meta-Review
Source: PLoS One. 2015 Jul 23;10(7):e0131448. doi: 10.1371/journal.pone.0131448 (PMC4512724; doi:10.1371/journal.pone.0131448)
Supplement: S1 Table — (DOCX) [file pone.0131448.s001.docx]

**Supporting information**

**Table** S**1 Full Medline search strategy**

1. Exp Self care/
2. Exp Communication/
3. Exp Professional Family Relations/
4. Exp Telephone/
5. Exp Professional Patient Relations/
6. Exp Health education/
7. Exp Attitude of health personnel/
8. Exp Cellular phone/
9. Exp Patient education as topic/
10. Exp Handheld computer/
11. Exp Self efficacy/
12. Exp Activities of Daily Living/
13. Exp Self help devices/
14. Exp Community health services/
15. Exp Rehabilitation/
16. (Self ADJ2 (car* or manag* or help or admistrat* or monitor* or medicat*)) or self-car* or self-manag* or self-help or self-adminisrat* or self-monitor* or self-medicat* or selfcar* or selfmanagement or selfhelp or selfadministrat* or selfmonitor* or selfmedicat* or SM.ti/ab.
17. Responsib* or Autonom*.ti/ab.
18. Manag* or copes or coping.ti/ab.
19. “Disease management”.ti/ab.
20. “expert patient”.ti/ab.
21. (professional or clinician) ADJ2 development.ti/ab.
22. Educat* or training or skill* or knowledge.ti/ab.
23. Confidence or self-efficacy.ti/ab.
24. (Access* or provi*) ADJ3 (information or records or results).ti/ab.
25. Monitor* or self-monitor* or selfmonitor*.ti/ab.
26. ((patient or individual* or person* or client*) ADJ3 (remind* or feedback)).ti/ab.
27. (Tele ADJ2 (health or medicine or care)) or tele-health or tele-medicine or tele-care or telehealth or telemedicine or telecare.ti/ab.
28. “Short message service” or SMS or “mobile phone” or “text message*”.ti/ab.
29. (home or environment* or living or assistive) ADJ2 (adaptation or modif* or equipment or technolog*).ti/ab.
30. “Care plan*”.ti/ab.
31. “Action plan*”.ti/ab.
32. Hypno* ADJ1 (self or home)ti/ab.
33. (cognitive or psychological or interpersonal or relaxation or biofeedback) ADJ3 (therap* or intervention* or program*).ti/ab
34. CBT.ti/ab.
35. Psychoeducation*.ti/ab.
36. (Peer or patient or emotional or social or psychosocial) ADJ1 (support or group) .ti/ab.
37. Financial ADJ1 control.ti/ab.
38. “personal health budget*”.ti/ab.
39. (Financial or monetary or payment* or discount or service*) ADJ5 incentiv*.ti/ab.
40. Exercise or training or rehabilitati*.ti/ab.
41. (Lifestyle or occupational) ADJ1 (intervention* or modification* or therapy) .ti/ab.
42. “Speech and language therapy”.ti/ab.
43. Or/1-42
44. Stroke/
45. Brain ischemia/
46. (Stroke or poststroke or post-stroke or cerebrovascu* or “brain vasc*” or “cerebral vasc*” or cva* or apoplexy* or sah) .ti/ab.
47. ((brain* or Cerebr* OR vascular OR cerebell* or intracran* or intracerebral* or subarachnoid) ADJ1 (accident OR isch?mi* OR infarct* or thrombo* or emboli* or occlus* or h?morrhage or h?matoma* or bleed*)).ti/ab.
48. Or/44-47
49. meta-analysis/
50. meta analysis as topic/
51. Review literature as topic/
52. MEDLINE.ti/ab.
53. (systematic review* or meta-analy* or metaanaly* or "research synthesis" or literature review) .ti/ab.
54. systematic ADJ3 literature.ti/ab.
55. data ADJ2 extract*.ti/ab.
56. ((information or data) ADJ3 synthesis).ti/ab.
57. Cochrane.ti/ab.
58. (qualitative or narrative or thematic or evidence or realist or interpret* or induct* or refutational or framework or systematic or textual) adj2 (approach or review* or synthes* or meta-summary or “meta summary” or summary).ti/ab.
59. Meta adj1 (summary or narrative or synthesis or ethnograph* or study or data or interpretation or aggregation or needs-assessment or “needs assessment”).ti/ab.
60. meta-summary or meta-narrative or meta-synthesis or meta-ethnograph* or meta-study or meta-data-analysis or meta-data-synthesis or meta-interpretation or meta-aggregation
61. “reciprocal translational analysis”.ti/ab.
62. RTA.ti/ab.
63. “lines-of-argu?ment synthesis” or “lines of argu?ment synthesis”.ti/ab.
64. “LOA synthesis”.ti/ab.
65. “grounded formal theory”.ti/ab.
66. “grounded theory synthesis”.ti/ab.
67. ecological adj2 (triangulation or sentence or synthesis).ti/ab.
68. Phenomenography.ti/ab.
69. ((mixed or multi* or cross) adj1 (method* or design* or research or strategy)) adj2 (synthesis or review).ti/ab.
70. (mixed-method* or multi-method* or mixed-design or multi-design or multiple-methods or multi-strategy or cross-design) adj2 (synthesis or review).ti/ab.
71. “research synthesis”.ti/ab.
72. Data ADJ2 extract*.ti/ab.
73. ((information or data) ADJ3 synthesis).ti/ab.
74. Bayesian adj1 (meta-analysis or “meta analysis”).ti/ab.
75. “case survey”.ti/ab.
76. “qualitative comparative analysis”.ti/ab.
77. Or/49-76
78. letter.pt
79. comment.pt
80. editorial.pt
81. Or/78-80
82. 77 not 81
83. 43 and 48 and 82

NB Search strategy for systematic reviews of RCTs was combined with a concurrent search of systematic reviews of qualitative work
