# Supplementary material for: Self-Management Support Interventions for Stroke Survivors: A Systematic Meta-Review
Source: PLoS One. 2015 Jul 23;10(7):e0131448. doi: 10.1371/journal.pone.0131448 (PMC4512724; doi:10.1371/journal.pone.0131448)
Supplement: S2 Table — (DOCX) [file pone.0131448.s002.docx]

**Supporting information**

**Table S2 R-AMSTAR criteria for quality assessment.^i^**

1. Was an a priori design provided?
2. Was there duplicate study selection and data extraction?
3. Was a comprehensive literature search performed?
4. Was the status of publication (i.e. grey literature) used as an inclusion criterion?
5. Was a list of studies (included and excluded) provided?
6. Were the characteristics of the included studies provided?
7. Was the scientific quality of the included studies assessed and documented?
8. Was the scientific quality of the included studies used appropriately in formulating conclusions?
9. Were the methods used to combine the findings of studies appropriate?
10. Was the likelihood of publication bias (a.k.a. “file drawer” effect) assessed?
11. Was the conflict of interest stated?
12. Kung J, Chiappelli F, Cajulis OO, et al. From Systematic Reviews to Clinical Recommendations for Evidence-Based Health Care: Validation of Revised Assessment of Multiple Systematic Reviews (R-AMSTAR) for Grading of Clinical Relevance. *Open Dent J* 2010;**4**:84-9
