# Supplementary material for: Self-Management Support Interventions for Stroke Survivors: A Systematic Meta-Review
Source: PLoS One. 2015 Jul 23;10(7):e0131448. doi: 10.1371/journal.pone.0131448 (PMC4512724; doi:10.1371/journal.pone.0131448)
Supplement: S3 Table — (DOCX) [file pone.0131448.s003.docx]

**Supporting information Table S3 Number of overlapping RCTs between systematic reviews**

|  | ***Aziz 2008*** | ***Hoffman 2010*** | ***Legg 2006*** | ***OST 2003*** | ***Poulin 2012*** | ***Steultjens 2003*** | ***Walker 2004*** | ***Ellis 2010*** | ***Ko 2010*** | ***Korpershoek 2011*** | ***Lui 2005*** | ***Rae-Grant 2011*** | ***Smith 2008*** |
| --- | --- | --- | --- | --- | --- | --- | --- | --- | --- | --- | --- | --- | --- |
| ***Aziz, 2008*** | *5* |  |  |  |  |  |  |  |  |  |  |  |  |
| ***Hoffman, 2010*** | 0 | *1* |  |  |  |  |  |  |  |  |  |  |  |
| ***Legg, 2006*** | 0 | 0 | *9* |  |  |  |  |  |  |  |  |  |  |
| ***OST, 2003*** | 0 | 0 | 7 | *14* |  |  |  |  |  |  |  |  |  |
| ***Poulin, 2012*** | 0 | 0 | 0 | 0 | *3* |  |  |  |  |  |  |  |  |
| ***Steultjens, 2003*** | 0 | 1 | 6 | 6 | 0 | *18* |  |  |  |  |  |  |  |
| ***Walker, 2004*** | 0 | 0 | 7 | 8 | 0 | 6 | *8* |  |  |  |  |  |  |
| ***Ellis, 2010*** | 0 | 0 | 0 | 1 | 0 | 0 | 0 | *16* |  |  |  |  |  |
| ***Ko, 2010*** | 0 | 0 | 0 | 0 | 0 | 0 | 0 | 0 | *0* |  |  |  |  |
| ***Korpershoek, 2011*** | 0 | 0 | 0 | 0 | 0 | 0 | 0 | 1 | 0 | *4* |  |  |  |
| ***Lui, 2005*** | 0 | 0 | 0 | 0 | 0 | 0 | 0 | 2 | 0 | 1 | *6* |  |  |
| ***Rae-Grant, 2011*** | 0 | 0 | 0 | 0 | 0 | 0 | 0 | 0 | 0 | 0 | 0 | *0* |  |
| ***Smith, 2008*** | 0 | 0 | 0 | 0 | 0 | 0 | 0 | 1 | 0 | 1 | 1 | 0 | *17* |
