# Supplementary material for: Self-Management Support Interventions for Stroke Survivors: A Systematic Meta-Review
Source: PLoS One. 2015 Jul 23;10(7):e0131448. doi: 10.1371/journal.pone.0131448 (PMC4512724; doi:10.1371/journal.pone.0131448)
Supplement: S4 Table — (DOCX) [file pone.0131448.s004.docx]

**Supporting information Table S4 Why the included systematic reviews are self-management support**

| **Review** | **Relevant aim(s)** | **Why this is self-management support** |
| --- | --- | --- |
| **Therapy rehabilitation: Interventions which are delivered by therapists (may include OTs and physiotherapists) working to rehabilitate individuals after a stroke** | | |
| **Aziz,**  **2008** | To determine whether therapy-based rehabilitation services influence stroke patient and carer outcomes one year or more after the index stroke, and which outcomes are influenced. | “All trials showed a similar approach that was based on a problem-solving method as a focus of intervention. This may suggest that, although each trial was different in design and methodology, the nature of the rehabilitation intervention itself shared a common aim, that is to reduce the level of disability by altering task-orientated behaviour and goal-orientated activities.” |
| **Hoffman,**  **2010** | To determine the effectiveness of occupational therapy for people with cognitive impairment after a stroke in improving functional performance of basic and instrumental ADL and cognitive abilities. | “These interventions may take either a remedial or a compensatory approach, or both. The remedial approach focuses on training specific cognitive deficits using media such as pencil and paper, computer tasks and board games. In a compensatory approach interventions may include (1) training skills for daily activities and vocation using compensatory strategies; (2) advising and educating about the use of assistive devices that aid cognitive function; and (3) educating patients, families, and caregivers about strategies to overcome patients’ cognitive impairment. The dynamic interactional approach is an integrated approach, encompassing both remedial and compensatory elements to encourage generalisation of the treatment effect achieved in a clinical setting to patients’ real life performance situation” |
| **Legg,**  **2006** | To determine whether any intervention provided by an occupational therapist (or under the supervision of an occupational therapist) with the specific aim of facilitating personal activities of daily living improves the outcomes for patients following stroke. | “Occupational therapy aims to enable people to achieve health, well being and life satisfaction through participation in occupation. Occupational therapy specifically aims to promote recovery through the use of purposeful activities... Occupational therapy interventions required to be focused on practice of personal activities of daily living or targeted towards improving the patient’s ability to perform personal activities of daily living.” |
| **OST,**  **2003** | To determine whether therapy-based rehabilitation services influence stroke patients and carer outcomes, and which outcomes are influenced. Further, to examine which components of therapy-based rehabilitation services are effective. | “Therapy rehabilitation (is) provided by physiotherapy, occupational therapy, or multidisciplinary staff working *with* patients primarily to improve task-orientated behaviour and hence reduce disability.”  “The definition of rehabilitation is broad and nonspecific: ’a problem-solving and educational process aimed at reducing the disability and handicap experienced by someone as a result of a disease ...’. Using this definition, outpatient stroke rehabilitation services can be considered as any intervention delivered by rehabilitation personnel, which aims to meet these broad objectives. These interventions have been categorised into those which aim to reduce disability and those aiming to reduce psychological and social problems.” |
| **Poulin,**  **2012** | To determine whether executive function intervention are more effective than no intervention or an alternative intervention in improving executive functions and functional abilities in daily life in the acute, sub-acute, and chronic stages of stroke recovery. | “Interventions that were offered individually or in groups and that involved components such as computerized cognitive training, problem solving, and strategy formation techniques, goal management training, or other compensatory strategies and external aids for overcoming everyday executive problems were all considered.” |
| **Steultjens,**  **2003** | To determine whether OT interventions improve outcome for stroke patients. | “Occupational therapy aims at facilitating task performance by improving relevant performing skills or developing and teaching compensatory strategies to overcome lost performance skills. Training of self-care activities, training of leisure activities, and advice and instruction regarding assistive devices are the 3 most frequently chosen interventions for stroke patients. In addition, the occupational therapist educates and shares information with the family and primary caregiver about the patient’s ability to perform and about how to provide proper assistance.” |
| **Walker,**  **2004** | To address the efficacy of community OT using individual patient data from randomised controlled trials. | “Occupational therapy is an essential component in the rehabilitation of stroke patients and is primarily concerned with the re-ablement and re-settlement of patients into their chosen home environment.” |
| **Other SM Support: A heterogeneous group of interventions delivered by various persons and of various modalities which aim to support stroke survivors / caregivers** | | |
| **Ellis,**  **2010** | To determine the efficacy of stroke liaison workers for patients with stroke and their caregivers in increasing participation and improving wellbeing for patients and carers. | “A stroke liaison worker can be defined as someone whose aim is to increase participation and improve wellbeing for patients and carers. Typically they provide emotional and social support and information to stroke patients and their families and liaise with services with the aim of improving aspects of participation and quality of life for patients with stroke, their carers, or both.” |
| **Ko,**  **2010** | To determine whether in patients with chronic disease, a patient-held medical record, compared to usual care, improves clinical care, patient outcomes or satisfaction. | “Patient-held medical records, where the patient is given a copy of the record to keep, and to take to health appointments, to help manage healthcare tasks and communication. PHRs are formal and structured records that are given to patients to enable the continuity and quality of care. They... include sections containing key patient and healthcare information, and usually contain blank sections to enable patient note-taking and healthcare staff notes.” |
| **Korpershoek, 2011** | To determine which self-efficacy enhancing interventions influence mobility, ADL, depression and HRQL of patients with a stroke. | “The concept self-efficacy is described as the confidence in one’s ability to perform a task or specific behaviour. A high sense of self-efficacy leads to desired outcomes, such as improved health. Self-efficacy is a situation- and task-related, behaviour specific concept.” |
| **Lui,**  **2005** | To examine the effectiveness of teaching problem solving skills to caregivers in stroke care. | “Teaching family caregivers to cope with problems and to relieve their own stress is essential, and there is some evidence that their well-being affects the health and recovery of stroke patients...Several problem solving strategies were examined and evaluated, including the use of positive problem orientation confronting or facing the problem, analyzing the problem and undertaking possible solutions, and goal setting.” |
| **Rae-Grant, 2011** | To review the current body of evidence supporting the efficacy of self-management programs in individuals with multiple sclerosis (MS) and other chronic neurological conditions. | “The efficacy of self-management programs in individuals with multiple sclerosis and other chronic neurological conditions. (Defined as) collaboratively helping patients and families acquire the skills and confidence to manage their chronic illness, providing self management tools, and routinely assessing problems and accomplishments.” |
| **Smith,**  **2008** | To examine the effectiveness of information strategies provided with the intention of improving the outcome for stroke patients or their identified caregivers or both. | “Information strategies provided with the intention of improving the outcome for stroke patients or their identified caregivers or both.” |
