# Supplementary material for: Self-Management Support Interventions for Stroke Survivors: A Systematic Meta-Review
Source: PLoS One. 2015 Jul 23;10(7):e0131448. doi: 10.1371/journal.pone.0131448 (PMC4512724; doi:10.1371/journal.pone.0131448)
Supplement: S5 Table — (DOCX) [file pone.0131448.s005.docx]

**Supporting information Table S5 Detailed R-AMSTAR results**

|  | ***Therapy rehabilitation*** | | | | | | | ***Other SMS*** | | | | | |
| --- | --- | --- | --- | --- | --- | --- | --- | --- | --- | --- | --- | --- | --- |
| **R-AMSTAR Criteria** | **Aziz, 2008** | **Hoffman. 2010** | **Legg, 2006** | **OST, 2003** | **Poulin, 2012** | **Steultjens, 2003** | **Walker, 2004** | **Ellis, 2010,** | **Ko, 2010** | **Korpershoek, 2011** | **Lui, 2005** | **Rae-Grant, 2011** | **Smith, 2008** |
| **Was an ‘a priori’ design provided?** | 4 | 4 | 4 | 4 | 4 | 4 | 3 | 4 | 4 | 4 | 2 | 3 | 4 |
| **Was there duplicate study selection and data extraction?** | 4 | 4 | 4 | 4 | 1 | 3 | 4 | 3 | 1 | 3 | 3 | 4 | 4 |
| **Was a comprehensive literature search performed?** | 4 | 4 | 4 | 4 | 4 | 3 | 4 | 4 | 3 | 2 | 3 | 3 | 4 |
| **Was the status of publication (ie grey literature) used as an inclusion criterion?** | 4 | 4 | 4 | 4 | 3 | 3 | 4 | 4 | 2 | 2 | 2 | 2 | 4 |
| **Was a list of studies (included and excluded) provided?** | 3 | 4 | 4 | 4 | 4 | 4 | 1 | 4 | 2 | 1 | 1 | 1 | 4 |
| **Were the characteristics of the included studies provided?** | 4 | 4 | 4 | 4 | 3 | 1 | 4 | 3 | 3 | 2 | 4 | 2 | 4 |
| **Was the scientific quality of the included studies assessed and documented?** | 4 | 4 | 4 | 4 | 3 | 3 | 4 | 3 | 4 | 2 | 1 | 3 | 4 |
| **Was the scientific quality of the included studies used appropriately in formulating conclusions?** | 4 | 1 | 4 | 3 | 4 | 4 | 2 | 3 | 4 | 3 | 4 | 4 | 4 |
| **Were the methods used to combine the findings of the studies appropriate?** | 3 | 3 | 3 | 3 | 3 | 4 | 3 | 4 | 4 | 1 | 1 | 1 | 3 |
| **Was the likelihood of publication bias assessed?** | 3 | 1 | 4 | 4 | 1 | 1 | 4 | 1 | 1 | 1 | 2 | 1 | 3 |
| **Was the conflict of interest stated?** | 3 | 2 | 3 | 3 | 2 | 2 | 2 | 2 | 3 | 3 | 1 | 3 | 2 |
| **Total score /44** | **40** | **35** | **42** | **41** | **32** | **32** | **35** | **35** | **31** | **24** | **24** | **27** | **40** |
